# Information exchange networks for chronic illness care in primary care practices: an observational study

**DOI:** 10.1186/1748-5908-5-3

**Published:** 2010-01-22

**Authors:** Michel Wensing, Jan van Lieshout, Jan Koetsenruiter, David Reeves

**Affiliations:** 1 Scientific Institute for Quality of Healthcare, Radboud University Nijmegen Medical Centre, P.O. Box 9101, 6500 HB, Nijmegen, the Netherlands; 2 National Centre for Primary Care Development and Research, University of Manchester, UK

## Abstract

**Background:**

Information exchange networks for chronic illness care may influence the uptake of innovations in patient care. Valid and feasible methods are needed to document and analyse information exchange networks in healthcare settings. This observational study aimed to examine the usefulness of methods to study information exchange networks in primary care practices, related to chronic heart failure, diabetes and chronic obstructive pulmonary disease.

**Methods:**

The study was linked to a quality improvement project in the Netherlands. All health professionals in the practices were asked to complete a short questionnaire that documented their information exchange relations. Feasibility was determined in terms of response rates and reliability in terms of reciprocity of reports of receiving and providing information. For each practice, a number of network characteristics were derived for each of the chronic conditions.

**Results:**

Ten of the 21 practices in the quality improvement project agreed to participate in this network study. The response rates were high in all but one of the participating practices. For the analysis, we used data from 67 health professionals from eight practices. The agreement between receiving and providing information was, on average, 65.6%. The values for density, centralization, hierarchy, and overlap of the information exchange networks showed substantial variation between the practices as well as between the chronic conditions. The most central individual in the information exchange network could be a nurse or a physician.

**Conclusions:**

Further research is needed to refine the measure of information networks and to test the impact of network characteristics on the uptake of innovations.

## Background

Providing healthcare to patients with a chronic illness is an important challenge for health systems, and has major implications for health professionals' tasks, the organization of healthcare delivery, and the societal costs of healthcare [1]. Many patients with chronic illness receive healthcare in primary care settings. Large variations have been reported in the organisation and delivery of chronic illness care in primary care practices [2]. Understanding of the social factors that influence the uptake of clinical or organisational recommendations is, as yet, limited. For example, evidence that perceived team climate and organisational culture are associated with professional performance or health outcomes in primary care is inconsistent [3,4]. In this paper, we consider the structure of the information exchange networks in a primary care practice as a potential determinant of the uptake of recommendations for patient care.

Theory on diffusion of innovations predicts that specific characteristics of social networks are associated with the uptake of practices [5]. For example, connections of network members to relevant individuals outside the network help to signal the existence of specific recommendations for patient care. More particularly, the presence of individuals in a network who are also members of other networks ('boundary spanners') is expected to increase the likelihood that a recommendation becomes known to members of the network. It has been suggested that the presence of weak ties in a network is associated with uptake of recommendations, because individuals with weak ties are more likely to be connected to other networks [6]. Other research suggests, however, that having a centralized network position is associated with better transfer of knowledge [7,8].

Awareness of the existence of (new) knowledge, such as revised clinical recommendations or new organizational models for chronic illness care, is a necessary first step for the taking up of an innovation. But the innovation will only be implemented when this awareness is translated into (change of) individual behaviors. Networks that are dense and non-hierarchical in terms of information exchange may be better for the uptake of complex innovations, because they may provide credibility and legitimacy to the new practice [9]. The information exchange and associated interaction in dense, non-hierarchical networks could speed up collective behavior change through mechanisms such as social comparison and role modeling, although obviously the quality of the connections plays a role as well.

It is unclear whether these and other hypotheses on the uptake of innovations apply to healthcare. Social networks have mainly been studied outside the healthcare domain, with only a few studies focused on healthcare professionals. For example, a study in England found that clinical directors were embedded in relatively small densely connected networks (cliques), while nursing directors had a central position in a more hierarchical network [10]. Therefore nursing directors may be more adapted to gathering and dissemination information. A study of primary care partnerships in Australia found that independent staff played a crucial role in holding partnerships together [11]. A study in the United States showed that primary care physicians obtained information from colleagues with greater expertise and experience as well as colleagues who were accessible based on location and schedule [12].

With few previous applications, greater understanding is required of appropriate methodologies for collecting and analyzing social network data in primary care settings. In particular, efficient and effective ways for collecting reliable primary data about the relationships between the members of the network are required. A pilot study used data from ethnographic field notes to construct matrices that indicated how practitioners interacted [11]. Network characteristics, such as density and centralization, were determined for the two practices in the study. The study illustrated the approach very well, but the methods used were resource intensive and time consuming.

In the study presented here, we developed and tested a short, structured questionnaire to collect data on information exchange networks in primary care practice. We focused on chronic heart failure (CHF), chronic obstructive pulmonary disease (COPD), and diabetes. These conditions were chosen because primary care has an important role in delivering care for these conditions in the Netherlands, while previous research showed that clinical and organizational recommendations were not optimally implemented [13]. We had the following objectives. The first was to test the feasibility of the data collection method in primary care practices. This had two aspects--to establish that adequate response rates could be achieved, and to test the reliability of the data obtained about information exchange. The second objective was to examine whether the networks differed systematically between the three chronic diseases and between the practices in terms of a number of key network parameters. In the Netherlands, many quality improvement initiatives have focused on diabetes and COPD, and relatively few on CHF, hence some differences may be expected. Finally, we looked for variation in network parameters between practices for each of the three chronic conditions; the measurement of network parameters is only useful if practices can be shown to differ in these characteristics.

## Methods

### Study design and study population

We performed an observational study using a convenience sample of primary care practices. Our study was linked to an evaluation of a quality improvement project, focused on CHF, in Southern and Eastern parts of the Netherlands. The quality improvement project comprised of outreach visits to 21 general practices, provision of structured case registration forms for CHF patients, and telephone follow-up by the outreach visitor. The practices were invited separately to participate in this study on networking, and 13 practices agreed. Finally, ten practices participated. The ethical committee Arnhem-Nijmegen waived approval for the quality improvement study, in which this study was embedded. The practices were seen as separate cases, each with their own information networks. All general practitioners (GPs), practice nurses, and practice assistants in the participating practices were invited to complete a structured questionnaire.

### Measures

We asked all health professionals in the practices about giving and receiving information around three chronic diseases: CHF, COPD, and diabetes. A written one-page questionnaire was developed (Additional File 1). This questionnaire listed the health professionals in a practice by name (GPs, practice nurses, practice assistants), and a number of types of health professionals outside the practice (designated by discipline only: other GPs, other practice nurses, cardiologists, internists, physiotherapists, and a category 'others'). We asked each health professional to report on information exchange with each listed person, for each of the three chronic conditions separately, and for giving and receiving information separately. A simple tick box response format to indicate 'yes' was used. The information being exchanged might concern individual patients, practice management, or treatment in general.

### Data-analysis

Response rates per practice were determined and descriptions of the information networks were made for each practice in terms of connections for receiving information within the practice and from healthcare providers outside the practice. We used UCINET 6 for the network analyses and SPSS15 for other analyses.

Reliability was determined by examining to what degree connections defined by receiving information were confirmed by those defined by providing information (simple matching) [14]. A 'match' of receiving and providing information between two professionals was based on the mutual agreement of either presence or absence of such connection. We did not expect complete agreement, as individuals may have different perceptions on the same communication process, but we expected a reasonable degree of similarity between receiving and providing information.

Next, we computed a number of key parameters of the networks of the practices, which we theorised could be predictive of the uptake and sustainable adoption of new practices. We based these calculations on the network of receiving information links, because we assumed that these were most crucial for the uptake of innovations. A non-technical description of the network parameters is provided:

Density-The density in a practice is the proportion of all possible connections in a network that are actually present. In a practice with a dense network, (new) information can flow directly between most individuals so that both the information is quickly shared as well as processes of interpretation and legitimization of the information are shared. This will result in a (often implicit) shared decision on how to act on the information.

Centralization-This is a measure for the degree that a network is organized around a single person. If one person gives information to all the other individuals in the network, the outdegree of centralization of the network is high. A high indegree of centralization indicates that information from many practice members flow to one person. In a practice network with high centralization, it is important to get the central individual involved in efforts to implement knowledge in routine healthcare delivery. This individual may be recognized as a local opinion leader.

Hierarchy-This is a measure for the direction in which information flows (note that it is not necessarily related to power). In a network without reciprocity, all information goes in one direction and the hierarchy will be strong. If the flow of information has two directions, there is a possibility for feedback and the hierarchy is lower. When the hierarchy of a network is low, more individuals in the practice can give information to other practice members. In a low hierarchy information exchange network, it is important to involve all members of the network in efforts to implement knowledge instead of targeting just specific individuals.

Overlap-The total overlap indicates the proportion of present and absent ties in an index network (of all that could exist) that also exist in another network. A high number of absent connections can result in high total overlap, therefore a second measure of overlap is the overlap in connected individuals. This measure is the total number of connections in two (or more) networks divided by the total number of individuals who are connected (not including individuals in a network which are not connected). It is the mean number of connections held by any individual in the networks, who has at least one connection. Overlapping information exchange networks in a practice, for example, regarding different chronic diseases, will enhance the speed of information exchange and likelihood of uptake in professional performance.

We substituted missing values in the information receiving networks by imputation from the information providing network, when available. If the response of an individual on receiving information was missing, it was substituted by the responses of the individuals who indicated they had provided information to this individual. This method is commonly used in social network analysis [15], although little is known about its appropriateness in the specific context of implementation research. We filled in a zero for no contact if both individuals did not provide information on their connection. Therefore, for further analysis a 'zero' in the data files referred to absence of a connection, or absence of data on presence of a connection.

We computed parameters thought to be associated with either learning about an innovation or the uptake of an innovation. Practice network parameters that may be related to learning about an innovation are: total number of external connections, number of external connections as a fraction of all connections, and proportion of external connections to the most central individual in the practice. Network characteristics that are potentially associated with actual uptake of the innovation are: density, centralization, hierarchy, and overlap between the three disease information exchange networks. Regarding centrality, we also determined the professional discipline (physician, nurse, assistant) of the individuals with the highest centralisation scores.

## Results

Ten of the 21 practices in the quality improvement project agreed to participate in our study on information exchange networks. Two of these ten participating practices consisted of one GP and one practice assistant; these practices were excluded from the analysis in this paper. Table 1 provides descriptive information on the information networks in the eight participating practices. Compared to the 21 practices in the quality improvement project, the participants in this networks study were less likely to be single-handed practices and practices without practice nurse. At the largest practice, ten out of the 20 practice staff (mostly practice assistants) did not complete the questionnaire. The number of connections for information exchange per condition varied between two and 47 within the practice (Table 1). On average, 65.6% of the receiving information connections (either presence or absence) were confirmed by the reported providing information connections. The agreement was lowest for the diabetes information networks in all but one practice.

**Table 1:** Numbers of health professionals and receiving information connections (n = 8 general practices)

Practice number	1	2	3	4	5	6	7	8	Total
Number of GPs	6	2	2	1	2	7	1	2	23

Number of assistants	7	3	4	2	2	9	2	3	32

Number of nurses	2	1	1	1	1	4	1	1	12

Total number of providers in the practice	15	6	7	4	5	20	4	6	67

Total number of non-responders*	0	0	0	1 (P)	2 (P, A)	10 (P,9A)	0	0	13

									

**Receiving information within the practice**									

Reported CHF connections	6	11	5	7	2	12	6	9	

Reported COPD connections	41	12	6	7	4	31	8	12	

Reported Diabetes connections	47	18	7	8	3	44	7	12	

Theoretical maximum number of present connections (n * (n - 1))	210	30	42	12	20	380	12	30	

									

**Proportion agreement between receiving and providing information**									**Mean**

CHF	0.948	0.567	0.810	0.667	1.00	0.864	0.833	0.767	0.807

COPD	0.919	0.733	0.667	0.667	1.00	0.833	0.667	0.867	0.794

Diabetes	0.862	0.667	0.619	0.500	0.833	0.689	0.417	0.867	0.682

*P = physician, N = nurse, A = assistant

Table 2 shows the values for density, centralization, and hierarchy of the information exchange networks (after imputation of missing values, where possible). Substantial variation existed between the practices as well between the chronic conditions. Density tended to be highest for diabetes and lowest for CHF, although two practices did not fit in this trend. Hierarchy of information exchange tended to have an opposite pattern to density, being lowest for diabetes and highest for CHF; three practices did not fit in this trend. Centralization (out degree and in degree) also showed high variation, but no clear pattern of differences emerged between the three conditions.

**Table 2:** Information receiving network characteristics

Practice		1 (n = 15)	2 (n = 6)	3 (n = 7)	4 (n = 4)	5 (n = 5)	6 (n = 20)	7 (n = 4)	8 (n = 6)
**Density**									

CHF		0.03	0.37	0.12	0.58	0.10	0.03	0.50	0.30

COPD		0.20	0.40	0.14	0.58	0.20	0.08	0.67	0.40

Diabetes		0.22	0.60	0.17	0.67	0.15	0.12	0.58	0.40

**Hierarchy**									

CHF		1.00	0.92	0.83	0.00	1.00	0.68	0.00	1.00

COPD		0.70	0.92	0.70	0.00	1.00	0.56	0.00	0.92

Diabetes		0.70	0.00	0.70	0.00	1.00	0.55	0.50	0.92

**Centralization**									

CHF	Outdegree %	12	76	25	56	19	24	67	84

	Indegree %	28	28	25	56	19	13	67	12

COPD	Outdegree %	71	72	22	56	28	63	44	72

	Indegree %	33	48	22	56	6	30	44	24

Diabetes	Outdegree %	83	48	39	44	13	54	56	72

	Indegree %	68	48	39	44	13	27	56	12

									

Professional discipline of individuals with highest outdegree centrality *									

CHF		N	P	P	P	P;N	P	P	P

COPD		N	P	P;N	P	N	P	P;N	P

Diabetes		P	P	N	P;N	P;N	P	N	P

* P = physician, N = nurse, A = assistant

**Figure 1:**
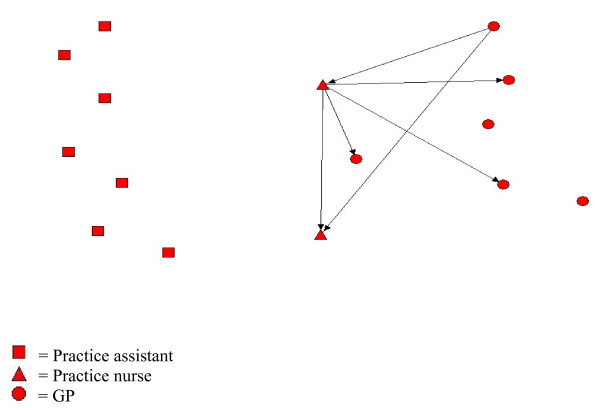
**Receiving information networks in practice one for chronic heart failure**. Visual presentation of information network of health professionals in practice one regarding chronic heart failre.

**Figure 2:**
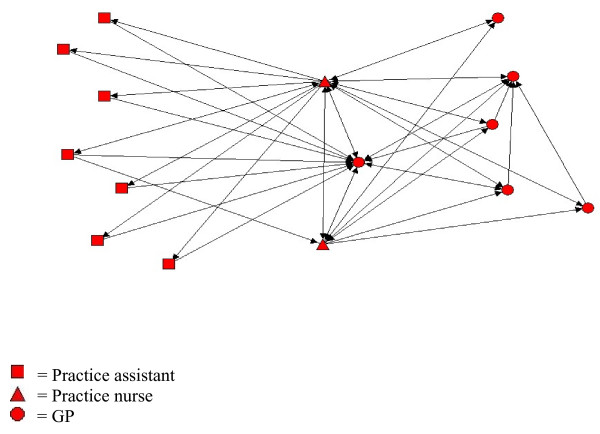
**Receiving information networks in practice one for diabetes**. Visual presentation of information network of health professionals in practice one regarding diabetes.

**Figure 3:**
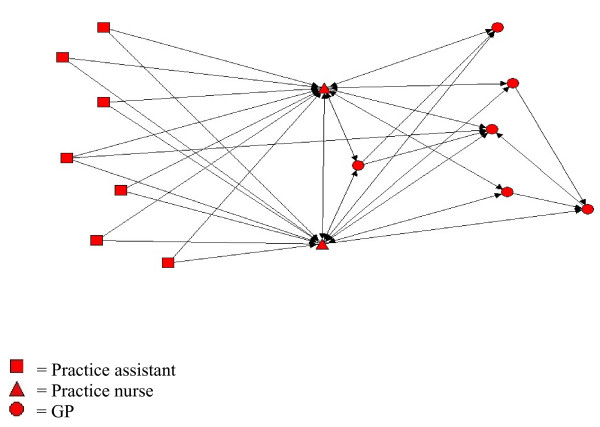
**Receiving information networks in practice 1 for COPD**. Visual presentation of information network of health professionals in practice one regarding COPD.

The professional discipline of the most central person(s) in a practice varied both across practices and between chronic conditions within practices. Within practice one, for example, care for COPD patients was centered around two nurses, to whom the practice assistants worked almost exclusively; whereas care for diabetic patients centered on a GP and one of these nurses, with the practice assistants again working almost entirely to these two individuals (Figures 1, 2, and 3). The role of practice assistants differed across the practices, reflecting the variation of clinical roles that these individuals have in general practices.

The overlap of information exchange connections across health conditions (CHF and COPD, CHF and diabetes, COPD and diabetes) is presented in Table 3. The overlap of (present or absent) connections was 80% or higher in all but one practice. This overlap was due to similarities in the absence of connections. Focusing on the similarities in presence of connections only, the mean number of connections amongst individuals with at least one connection varied substantially across practices and chronic diseases.

**Table 3:** Overlap between disease-specific information networks

		Total	Connected individuals
Practice 1	CHF-COPD	0.833	1.146

	CHF-Diabetes	0.805	1.128

	COPD-Diabetes	0.790	1.333

	CHF-COPD-Diabetes		1.529

			

Practice 2	CHF-COPD	0.967	1.917

	CHF-Diabetes	0.767	1.611

	COPD-Diabetes	0.800	1.667

	CHF-COPD-Diabetes		2.071

			

Practice 3	CHF-COPD	0.929	1.571

	CHF-Diabetes	0.905	1.500

	COPD-Diabetes	0.976	1.857

	CHF-COPD-Diabetes		2.250

			

Practice 4	CHF-COPD	1.000	1.000

	CHF-Diabetes	0.917	1.875

	COPD-Diabetes	0.917	1.875

	CHF-COPD-Diabetes		2.750

			

Practice 5	CHF-COPD	0.900	1.500

	CHF-Diabetes	0.950	1.667

	COPD-Diabetes	0.950	1.750

	CHF-COPD-Diabetes		2.250

			

Practice 6	CHF-COPD	0.918	1.188

	CHF-Diabetes	0.889	1.200

	COPD-Diabetes	0.887	1.192

	CHF-COPD-Diabetes		1.370

			

Practice 7	CHF-COPD	0.833	1.750

	CHF-Diabetes	0.417	1.300

	COPD-Diabetes	0.583	1.500

	CHF-COPD-Diabetes		2.100

			

Practice 8	CHF-COPD	0.90	1.818

	CHF-Diabetes	0.90	1.818

	COPD-Diabetes	1.00	2.000

	CHF-COPD-Diabetes		2.818

The number of connections to healthcare providers outside the practice varied from two to 15 per chronic condition (Table 4). The most central individual in the network (as defined by internal information exchange network in the practice) often had less than one-half of the connections to individuals outside the practice, indicating that the majority of the information receiving connections to external professionals were distributed among individuals less central in the internal information exchange networks.

**Table 4:** Connections outside the practice

Practice	1 (n = 15)	2 (n = 6)	3 (n = 7)	4 (n = 4)	5 (n = 5)	6 (n = 20)	7 (n = 4)	8 (n = 6)
**Receiving information from ** **outside the practice**								

Reported CHF connections	3	7	3	4	2	2	2	5

Reported COPD connections	11	5	3	5	4	5	2	5

Reported Diabetes connections	14	6	5	4	6	15	2	6

								

**Percentage of outside connections of ** **all connections for the disease**								

CHF	33	39	25	44	50	18	25	46

COPD	21	29	16	50	57	17	20	36

Diabetes	23	25	19	44	75	32	22	40

								

**Number of outside connections hold by ** **the most central individual out ** **of all outside connections**								

CHF	0/3	1/7	1/3	0/4	2/2	0/2	2/2	3/5

COPD	4/11	1/5	1/3	4/5	2/4	1/5	2/2	2/5

Diabetes	2/14	1/6	0/5	2/4	2/6	3/15	0/2	2/6

## Discussion

This study showed that connections for exchange of information around specific chronic diseases could be measured with a simple structured questionnaire. About one-half the practices in a quality improvement project were willing to participate in this study of information exchange networks. The reliability of the data, in terms of receiving information confirmed by providing information, was reasonably high overall, but could be low in specific networks. Substantial variation across practices and chronic conditions was found regarding various network parameters. These results support undertaking further research to refine the measure and to examine associations between network characteristics and uptake of innovations in primary care practices.

Our study was done in a convenience sample of practices, focusing on providing 'proof of principle'. The results should not be translated to other settings, because the sample of practices was not representative of any larger group. We had a broad focus on information exchange that encompassed both information on individual patients and information on practice development. A more specific focus might change the study results. For example, another study in one large primary care practice used just one question, focused on women's health issues [12]. Our focus was on receiving information relationships, because we considered this most relevant for the uptake of innovations, but an alternative approach would be to focus on relationships with confirmed ties (both receiving and providing information). Further validation of the measure used could focus on confirmation of the reported connections by other measures, such as analysis of patient records or direct observation in the practice. Another area for development is more detailed identification and analysis of links to health professionals outside the practice, which was only of secondary interest in this study.

Previous network studies in healthcare have not fully reported on participation and response rates [11,12]. In our study, about one-half of the practices we approached participated in the networks study. This may suggest problems with the feasibility of network studies in healthcare settings. It should be noted that the practices were already participating in a quality improvement project, which may have affected recruitment to this study. Recruitment for network studies is an area for further research. The handling of missing values is a particularly difficult aspect of network analysis [15]. Simulation studies have suggested that response rates of 70% to 80% are required to derive reliable estimates of many network parameters [15]. Our study achieved reasonably high response rates, except in one large practice. This practice reported problems with the interpretation of the form. Most practices in this study did not have many staff, and it is possible that larger practices will not provide such high response rates, particularly as the network data collection form increases in length with the size of the practice.

Patterns in the practice scores on the network characteristics support the face validity of the method. For example, the dense information networks for diabetes and COPD may reflect the fact that in the Netherlands many practice nurses and supportive staff have a recognized role in providing patient care for these conditions, as opposed to CHF. It may also reflect the stronger focus on diabetes and COPD, compared to CHF, in nationwide programmes for quality improvement in the Netherlands. The lower density of the CHF network in the practices may provide a challenge for the uptake of new clinical recommendations and models for structured chronic care. Such innovations may not be reinforced by the social influence mechanisms that are associated with dense networks, and therefore less likely to be implemented quickly. However, it is important to mention that social networks may function in counter-intuitive ways that may reduce the relevance of perceived face validity. Furthermore, network characteristics that were not studied, such as 'trust' and 'tie strength', have been found to enhance the uptake of innovations in non-healthcare settings [7]. Empirical and analytical research is needed to identify the social network processes that facilitate knowledge transfer and uptake of innovations.

Information from people outside the practice can come through various individuals into the practice. These connections, through which innovations may be introduced into a practice, were clustered to some extent in the most central individuals in the internal information exchange networks. This might enhance the uptake of innovations, because a centralized position in a network has been found to be associated with knowledge transfer [7]. But even so, the majority of external connections were shared among less central individuals. Thus, while we found that the core individuals within the practice networks also tended to be the most prolific boundary spanners, information was also received through other channels. This may be important, because the adoption of an innovation is associated with the availability of multiple sources of information [9]. Further research is required to explore the role of various individuals in the information exchange in a practice with individuals outside the practice.

As many patients with chronic illness have several chronic conditions (multi-morbidity), it was relevant to observe that the information exchange networks within practices for the three chronic conditions showed overlap. Overlap suggests that patients with multi-morbidity receive care for each of their chronic conditions from very much the same set of individuals. We can conjecture that this will be associated with better integration of care, higher efficiency of service delivery, and more patient-centered care. Conversely, low overlap suggests that care for each condition is provided by quite different practice teams, with medical notes providing the main, or only, means of communication and coordination between teams.

The central individual in the information exchange networks could be a nurse or a physician, and in some practices this differed across the chronic conditions. This might reflect differences in the functioning of practices, which may be related to practice policies on how care is organised for particular conditions or to the presence of staff with particular skills or interests. We used formal network analysis to identify the central members of the network, but simple inspection of the network maps themselves can identify other particular types of individuals, such as those who are isolated from the network (*i.e.*, lack links to others), and 'brokers' who control the flow of information from one part of the network to another [5].

What does this study contribute to implementation science? While social network studies can be used to examine a wide variety of consequences and determinants of network configurations, our study concerned the potential impact of networks on uptake of (new) knowledge in clinical practice. We applied concepts and methods from 'diffusion of innovations' research and 'evidence-based medicine' research, two research traditions that have historically developed independently from each other [16]. Our study fits with calls to use theory-based approaches in research on the uptake of research findings [17]. It remains to be seen if social networks can be changed in ways that encourage the implementation of new knowledge is indeed enhanced. However, currently available implementation interventions targeted at individual health professionals (focused on their motivation and competence) have mixed, and on average moderate impact [18]. Therefore, there is a need for complementary methods that increase the impact of implementation interventions.

Using network analysis to promote the uptake of research knowledge is not an entirely new approach in evidence-based medicine. Previous studies used sociometric methods to identify local opinion leaders and involve them in the promoting of the uptake of interventions. For example, a study in Scotland showed that the feasibility of this approach was variable across different professional groups and settings [19]. In combination with professional education, the approach had mixed effects on professional performance [20]. Involving opinion leaders is just one intervention based on network analysis. Other network-based implementation interventions could be related to patient care teams, such as changes in the range of professional competencies included and their coordination structures [21]. Yet another set of interventions could be linked to health professionals' communities of practice, although the exact meaning and implications of these remain topic of debate [22]. Social networks analysis can provide the concepts and methods to operationalise such approaches, but more research is needed on the validity and feasibility of the method for this purpose.

## Summary

Further research is required to refine the measure of information networks and to look for possible effects of specific network characteristics and knowledge utilization in primary care practices. Insight into information networks in healthcare organizations adds to the body of literature on social networks and diffusion of innovations, which has focused on innovation in larger organizations [23]. If future research on information exchange networks in healthcare is fruitful, the method might inform the tailoring of interventions to a specific network to facilitate more effective and efficient knowledge utilization. Also, network data may be used directly to provide feedback to practices and stimulate reflection on working patterns in a practice in order to encourage organizational development.

## Competing interests

The authors declare that they have no competing interests.

## Authors' contributions

MW designed the study, coordinated data-analysis, and wrote the paper. JvL coordinated data collection and contributed to the paper. JK was responsible for data analysis and contributed to the paper. DR supervised data analysis and contributed to the paper. All authors read and approved the manuscript.

## Supplementary Material

Additional file 1 Questionnaire on information exchange. Click here for file
