# Supplementary material for: Information exchange networks for chronic illness care in primary care practices: an observational study
Source: Implement Sci. 2010 Jan 22;5:3. doi: 10.1186/1748-5908-5-3 (PMC2822738; doi:10.1186/1748-5908-5-3)
Supplement: Additional file 1 — Questionnaire on information exchange. [file 1748-5908-5-3-S1.DOC]

**Additional file 1: Questionnaire on information exchange**

Name

Profession

Practice ID

Please tick those who gave you information and whom you gave information on chronic heart failure, COPD and depression in the previous 12 months. This information may concern individual patients, practice management, or treatment in general.

|  | | Chronic heart failure | | COPD | | Diabetes | |
| --- | --- | --- | --- | --- | --- | --- | --- |
| I gave or received information | | Gave | Received | Gave | Received | Gave | Received |
| Staff in your practice | |  |  |  |  |  |  |
| GP1 | Names |  |  |  |  |  |  |
| GP2 |  |  |  |  |  |  |
| GP3 |  |  |  |  |  |  |
| GP4 |  |  |  |  |  |  |
| Practice assistant 1 |  |  |  |  |  |  |
| Practice assistant 2 |  |  |  |  |  |  |
| Practice assistant 3 |  |  |  |  |  |  |
| Nurse 1 |  |  |  |  |  |  |
| Nurse 2 |  |  |  |  |  |  |
| Nurse 3 |  |  |  |  |  |  |
| **People outside your practice** | |  |  |  |  |  |  |
| Other GPs | |  |  |  |  |  |  |
| Other practice assistants | |  |  |  |  |  |  |
| Other nurses | |  |  |  |  |  |  |
| Dietician | |  |  |  |  |  |  |
| Physiotherapist | |  |  |  |  |  |  |
| Psychologist | |  |  |  |  |  |  |
| Medical specialist: Cardiologist | |  |  |  |  |  |  |
| Medical specialist: Pulmonologist | |  |  |  |  |  |  |
| Medical specialist: Internist | |  |  |  |  |  |  |
| Others, specify | |  |  |  |  |  |  |
